# 908. Dismantling Barriers to Hepatitis Elimination: Automated Hepatitis C Screening with Care and Cure by a Primary Care Based Team

**DOI:** 10.1093/ofid/ofab466.1103

**Published:** 2021-12-04

**Authors:** Binghong Xu, Ruth P Brogden, Ammie J Patel, Alyssa Gallipani, Jaymie Yango, Mary O Adedeji, Su Wang

**Affiliations:** 1 Center for Asian Health, RWJBH-Saint Barnabas Medical Center, Florham Park, New Jersey; 2 RWJBH-Saint Barnabas Medical Center, Florham Park, New Jersey; 3 Ernest Mario School of Pharmacy, Rutgers, the State University of New Jersey, Livingston, New Jersey; 4 Fairleigh Dickinson University School of Pharmacy, Eatontown, New Jersey; 5 RWJBH - Saint Barnabas Medical Center, Livingston, New Jersey; 6 Saint Barnabas Medical Center, Hillside, New Jersey; 7 Saint Barnabas Medical Center & World Hepatitis Alliance, New Providence, New Jersey

## Abstract

**Background:**

Liver cancer rates are rising in the US, viral hepatitis accounting for more than 65% of the cases. Yet more than half of viral hepatitis infections remain undiagnosed. In response to the rise in HCV due to the opioid epidemic, the Centers for Disease Control and Prevention began recommending a one-time HCV test for all adults in 2020. Screening, linkage to care (LTC) and access to HCV curative therapy must be scaled up to reach the WHO goal of eliminating hepatitis by 2030.

**Methods:**

In 2018, automated HCV screening utilizing electronic medical record protocols began in the emergency department (ED) based on the date of birth. Drug testing and peer recovery consults were added as eligibility criteria. Screening became universal and expanded to the inpatient units in 2020. Patient navigators (PN) received alerts of positive results and worked with patients to arrange LTC, one site being a primary care-based practice (PCP) where internists provided HCV care and support from ambulatory care clinical pharmacists.

**Results:**

From Mar 2018 to Mar 2021, 50,873 people were screened for HCV, with 977 (1.9%) testing HCV Ab+, and 259 (0.5%) had confirmed infection by reflex HCV RNA. LTC 86.6% of patients, and 128 (49.4%) were newly diagnosed. Universal screening led to 35,482 testings from Jan 2020-Mar 2021. People born out of the 1945-65 birth cohort made up 75.8% of the screened and 39.1% of the infected. The PCP evaluated 47 HCV patients, initiated therapy in 38; 36 required prior authorization and 15 needed financial assistance. Treatment breakdown was: 29 (76.3%) glecaprevir/pibrentasvir, 6 (15.8%) sofosbuvir/velpatasvir & 3 (7.9%) ledipasvir/sofosbuvir. Pharmacist intervention with prior authorizations and financial assistance significantly reduced the cost (table 1). Thus far, 35 achieved cure with undetectable HCV RNA at 12 weeks.

Table 1. The Cost of Treatment before and after Pharmacist Assistance

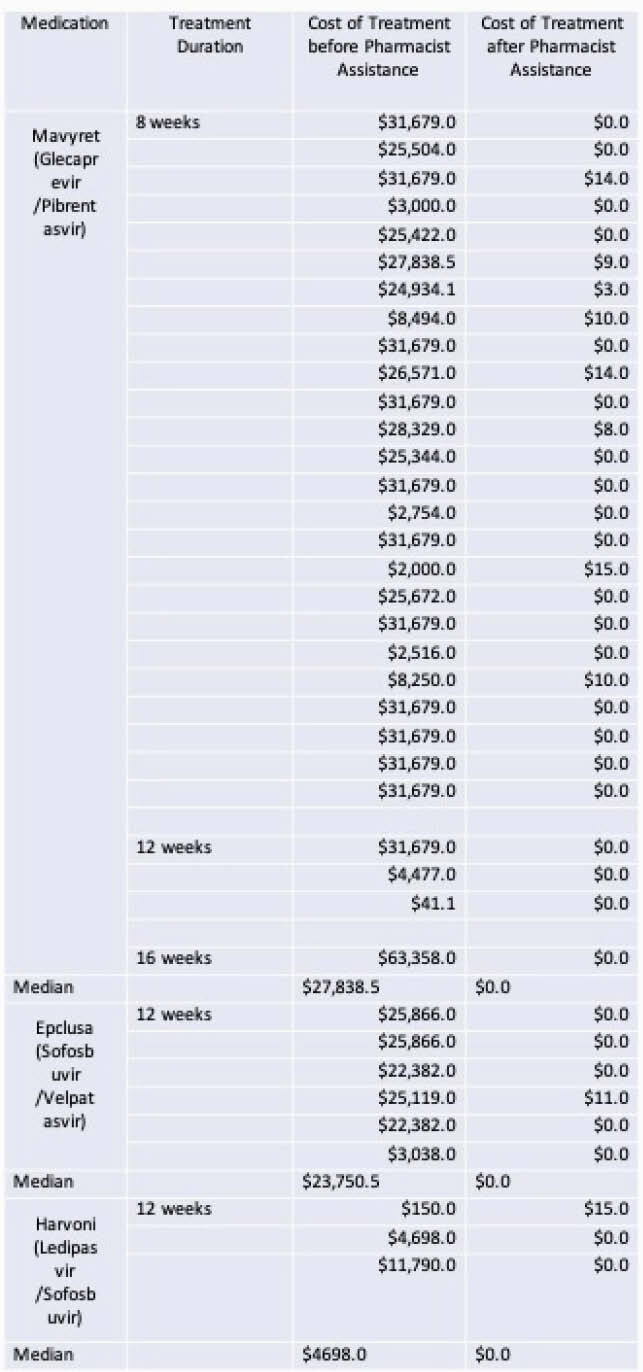

**Conclusion:**

Automated universal testing was an effective and seamless way to scale up HCV screening. Warm handoffs from a PN were important for engaging patients in care. A team approach assisted with removing barriers in therapy access, including prior authorization, specialist requirements, and financial assistance. Novel strategies utilizing ED and hospitals for testing with coordination to PCP are needed to find the missing millions and achieve hepatitis elimination.

**Disclosures:**

**Su Wang, MD MPH**, **Gilead Sciences** (Grant/Research Support)**Gilead Sciences** (Grant/Research Support)

